# Breaking the Cycle: Housing as a Key to Mental Health Recovery in Severe Mental Disorders

**DOI:** 10.1192/j.eurpsy.2026.11845

**Published:** 2026-07-31

**Authors:** M. Ligero Argudo, I. M. Peso Navarro, C. García Cerdán, C. Marín Lorenzo, R. A. Soto Limo, I. Rodríguez Blanco, C. Munaiz Cossio, P. Andrés Olivera

**Affiliations:** Psychiatry, Hospital Clínico de Salamanca, Salamanca, Spain

## Abstract

**Introduction:**

Social Determinants of Health established by the World Health Organization (WHO) include housing as a key determinant with direct impact in physical and mental well-being. Lack of adequate housing contributes to the onset and chronification of severe mental disorders. In its most extreme form, homelessness has been strongly associated with higher prevalence of disorders like substance use, psychotic and affective disorders. This vulnerable group also faces different obstacles and barriers in terms of access to habitual health resources and treatments.

**Objectives:**

- To examine the relationship between inadequate housing conditions and mental health, focusing on seve mental health disorders.
- To emphasize recent evidence on housing-based interventions and its effects in clinical and social outcomes.

**Methods:**

A descriptive observational studio was conducted using tools as PubMed, Medline, UpToDate and Scopus, including recent published studies. Systematic reviews, meta-analysis and observational studies were included, specially those assesing social determinants of health and housing conditions, homelessness and severe mental health disorders.

**Results:**

Recent evidence shows that people experiencing homelessness present significantly higher rates of severe mental health disorders compared to general population. Inestability in housing conditions is related to increased hospitalizations, clinical descompensations and higher morbility and mortality. Housing-based interventions such as Housing-First, associated with regular clinical support have shown better results in treatment adherence, hospitalization and social and general functioning.

**Conclusions:**

The relationship between housing and mental health is proven to be bidirectional: lack of housing triggers and exacerbates psychiatric deterioration, while mental health disorders increase housing vulnerability and inestability. Access to adequate housing should be considered as an essential intervention in public health and psychiatric practices as it increases the potential to improve the quality of life and decrease the social and healthcare costs associated with homelessness.

**Disclosure of Interest:**

None Declared

